# Supplementary material for: Improving statistical reporting in psychology
Source: Commun Psychol. 2025 Nov 14;3:156. doi: 10.1038/s44271-025-00356-w (PMC12618885; doi:10.1038/s44271-025-00356-w)
Supplement: Supplementary file 2 — Supplemental Material [file 44271_2025_356_MOESM2_ESM.pdf]

# Transparent Statistical Reporting in Psychology (TSRP) Checklist

This checklist is part of the companion materials to "*Improving Statistical Reporting in Psychology*" by Schubert et al (2025). It summarizes key recommendations for transparent reporting across the planning, analysis, and results stages of psychological research. While designed to accompany the examples in the paper, it can also be used independently as a practical guide for enhancing clarity, reproducibility, and interpretability in empirical work.

For each item, indicate the page number where it is addressed in the manuscript, or check "N/A" if not applicable.

| Section | Item | Page # | N/A |
|---|---|---|---|
| | **I. Planning Stage** | | |
| **Hypotheses and Design** | Hypotheses are clearly and precisely stated (directional, testable, falsifiable). | | ☐ |
| | Constructs are explicitly defined and operationalized. | | ☐ |
| | If applicable, formalized hypotheses (e.g., mathematical models) are provided. | | ☐ |
| **Preregistration** | Study preregistered with detailed methods, hypotheses, and analysis plan. | | ☐ |
| | Location and version of the preregistration (e.g., OSF URL) is cited. | | ☐ |
| | Deviations from preregistered plan are transparently documented. | | ☐ |
| **Sample Size Justification** | Justification for sample size is provided (e.g., a priori power analysis, precision goals). | | ☐ |
| | If simulation-based power analysis is used, code and assumptions are described/shared. | | ☐ |
| | Sequential design: Stopping rule, maximal sample size, and ASN rationale are stated. | | ☐ |
| | **II. Analysis Stage** | | |
| **Outliers and Missing Data** | Criteria for identifying outliers are pre-specified and reported. | | ☐ |
| | Proportion of outliers removed and rationale for removal are documented. | | ☐ |
| | Missing data mechanism is assessed (MCAR/MAR/MNAR). | | ☐ |
| | Missing data handling strategy (e.g., multiple imputation) is described and justified. | | ☐ |
| | Statistical models/tests are described in sufficient detail for replication. | | ☐ |

| | | | |
|---|---|---|---|
| **Statistical Model Specification** | Software, packages, and versions used are specified. | | ☐ |
| | Model assumptions are checked and violations are reported with remediation. | | ☐ |
| **Transparency & Reproducibility** | All code (or sufficient pseudocode) is provided via supplementary files or repository. | | ☐ |
| | Syntax files are exported from graphical user interface tools (e.g., SPSS, JASP). | | ☐ |
| | Simulated or real datasets are made available (when ethical and legal). | | ☐ |
| **III. Results Reporting** | | | |
| **Descriptive Statistics** | Means, medians, standard deviations, and sample sizes are reported for all variables. | | ☐ |
| | Data distributions are visualized using appropriate plots (e.g., violin, raincloud). | | ☐ |
| | Graphical visualizations are annotated and designed for interpretability. | | ☐ |
| **Inferential Statistics** | All statistical test results are fully reported: statistic, df, p-value, effect size, CI. | | ☐ |
| | Exact p-values are reported (not just p < .05). | | ☐ |
| | Confidence or credible intervals are provided and interpreted. | | ☐ |
| **Bayesian Analyses** | Priors are explicitly stated and justified. | | ☐ |
| | Bayes factors or posterior summaries are reported with interpretation. | | ☐ |
| | Prior sensitivity analysis results are included. | | ☐ |
| **Sequential Designs** | Type of sequential test and stopping rules are specified. | | ☐ |
| | Decision boundaries, sample sizes at each look, and final sample size are reported. | | ☐ |
| | Adjustments for bias or CI corrections are described. | | ☐ |
| **Null Results** | Null findings are contextualized with respect to power and precision. | | ☐ |
| | Equivalence testing is used where applicable; margins are justified and reported. | | ☐ |
| | Bayesian support for null hypotheses is quantified and interpreted cautiously. | | ☐ |

## Glossary

This glossary is part of the companion materials to *Improving Statistical Reporting in Psychology* by Schubert et al. (2025). Terms and definitions reflect the paper's usage and recommendations.

### A

**Alpha (α)**

Pre-specified Type I error rate used to define rejection regions in Neyman–Pearson testing.

**Alpha-spending function**

In group sequential designs, a rule that controls the cumulative Type I error across interim looks.

**Average sample number (ASN)**

The expected sample size required to reach a decision under a given sequential design and effect size.

### B

**Bayes factor ($BF_{10}$, $BF_{01}$)**

Ratio quantifying how much more likely the observed data are under one hypothesis or model than another (e.g., $BF_{10} = 5$ indicates data are five times likelier under $H_1$ than $H_0$).

**Bayesian inference / statistics**

A framework that combines prior information with data to produce posterior distributions; supports hypothesis testing via Bayes factors and estimation via posteriors and credible intervals.

**Beta (β)**

Type II error rate: the probability of failing to reject $H_0$ when $H_1$ is true; statistical power is $1 - \beta$.

**Beta-spending function**

An optional rule in group sequential designs to manage cumulative Type II error across interim looks.

**Bar chart (descriptives)**

Aggregated bars for counts or proportions; for continuous outcomes, bar charts of means (±SE/SD) obscure distribution shape and can mislead. Prefer box/violin/raincloud when describing distributions.

**Box plot (descriptives)**

Summarizes a distribution via median, interquartile range (box), and whiskers (e.g., 1.5×IQR), with optional outliers; conveys spread/skew more transparently than bar charts for continuous data.

**C**

**Complete case analysis (list-wise deletion)**

Analyzing only cases with no missing values; acceptable under MCAR but otherwise discouraged due to bias and loss of precision.

**Confidence interval (CI)**

A frequentist interval that contains the true parameter in a given proportion of repeated experiments (e.g., 95% in the long run); not a probability statement about the parameter.

**Credible interval**

A Bayesian interval giving the range within which the parameter lies with a stated probability, conditional on the prior and the data.

**D**

**Decision boundary (sequential designs)**

A pre-specified threshold (e.g., Bayes factor or likelihood ratio) for stopping data collection for efficacy or futility.

**E**

**Equivalence margin**

Predefined bounds that represent effects too small to be practically meaningful; equivalence is concluded when the entire CI lies within these margins.

**Equivalence testing**

Assesses whether an effect is small enough to be considered practically negligible (e.g., via the TOST procedure or a 90% CI at $\alpha = .05$).

**F**

**Fixed-sample design**

A design with final sample size set in advance; unplanned interim looks with p-value inference inflate Type I error (optional stopping).

**G**

**Group sequential design (GSD)**

A frequentist sequential framework with a maximum N and planned interim looks; uses alpha-spending to control Type I error.

**H**

**HARKing (Hypothesizing After the Results are Known)**

Post hoc framing of exploratory findings as confirmatory; misrepresents the research process and inflates familywise Type I error.

**M**

**Manipulation check**

An empirical test confirming that an experimental manipulation changed the intended construct.

**MAR / MCAR / MNAR**

Missingness mechanisms—*Missing Completely At Random* (unrelated to observed or unobserved data), *Missing At Random* (depends on observed data), *Missing Not At Random* (depends on unobserved data).

**Minimal / Smallest effect size of interest (SESOI)**

The smallest effect worth detecting, defined on theoretical or practical grounds and used for planning and interpretation.

**Missing data**

Unobserved values for otherwise relevant variables; recommended treatments include multiple imputation or full-information maximum likelihood rather than list-wise deletion (except under MCAR).

**Monte Carlo power simulation**

A simulation-based approach to estimate power and sample size, especially for complex models.

**Multiverse analysis**

Assessing robustness by running a principled set of plausible preprocessing and modeling pipelines rather than committing to a single path.

**O**

**Optional stopping**

Inspecting results and stopping early in fixed-sample frequentist designs; inflates Type I error with p-value inference. Less problematic in some Bayesian settings but debated.

**P**

**p-value**

The probability of data at least as extreme as observed, assuming $H_0$ and its model are true; not the probability that $H_0$ is true.

**Posterior distribution**

The updated probability distribution over parameters after observing data and combining with priors.

**Preregistration**

A time-stamped specification of hypotheses, design, and analysis plans prior to data collection/analysis to constrain researcher degrees of freedom and to clarify confirmatory vs. exploratory work.

**Prior distribution**

A quantified belief about parameters before seeing the current data; should be stated and, ideally, subjected to sensitivity analysis.

**Q**

**Questionable research practices (QRPs)**

Flexible or selective analyses/reporting that inflate effect sizes and reduce replicability, complicating sample-size planning.

**R**

**Raincloud plot**

A visualization combining raw data, distribution, and summary that conveys distributional shape better than bar charts for descriptive reporting.

**Robust estimators / bootstrapping**

Methods (e.g., M-estimators, trimmed means, resampling) that reduce undue influence of outliers while preserving data integrity.

## S

### Sample-size planning (frequentist)

An a priori determination of N based on α, desired power (1 − β), the effect size under $H_1$, and the planned test.

### Safeguard power analysis

A planning approach that uses a lower-bound effect size (accounting for estimate uncertainty or publication bias) to avoid underpowered designs.

### Sequential Bayes factor (SBF)

A sequential test that computes Bayes factors during data collection and stops when pre-set BF thresholds are crossed.

### Sequential design

A planning strategy (frequentist or Bayesian) allowing interim looks and early stopping per prespecified criteria to increase efficiency.

### Sequential Probability Ratio Test (SPRT)

A likelihood-ratio–based sequential test with flexible stopping; often more efficient than fixed-N tests.

### Statistical power (1 – β)

The long-run probability that a test will lead to rejection of $H_0$ when a specified $H_1$ is true.

## T

### Transparent Statistical Reporting in Psychology (TSRP) Checklist

A companion checklist aligning with the paper's guidance for planning and reporting analyses.

### Type I error (α) / Type II error (β)

False positive and false negative rates controlled within the Neyman–Pearson framework.

### Two One-Sided Tests (TOST)

An equivalence testing procedure that rejects both one-sided nulls to declare the effect practically equivalent to zero within set bounds.

## V

### Violin plot (descriptives)

Kernel-smoothed density (mirrored) that visualizes the full distribution, often combined with a box or median marker; provides more distributional detail than bar charts. Use with raw data overlays (e.g., raincloud) when feasible.
